# Self-antigen MASH2 combined with the AS15 immunostimulant induces tumor protection in colorectal cancer mouse models

**DOI:** 10.1371/journal.pone.0210261

**Published:** 2019-01-25

**Authors:** Clément R. Rioux, Margie L. Clapper, Harry S. Cooper, Jean Michaud, Natalie St Amant, Hossein Koohsari, Laura Workman, Esther Kaunga, Harvey Hensley, Anthony Pilorget, Catherine Gerard

**Affiliations:** 1 GSK, Laval, QC, Canada; 2 Fox Chase Cancer Center, Philadelphia, PA, United States of America; 3 GSK, Rixensart, Belgium; Howard University, UNITED STATES

## Abstract

Human achaete scute homolog 2 (HASH2) and its murine ortholog MASH2 are potential targets for colorectal cancer immunotherapy. We assessed immunogenicity and antitumor potential of recombinant MASH2 protein combined with AS15 immunostimulant (recMASH2+AS15) in CB6F1 and *Apc*^*+/Min-FCCC*^ mice. CB6F1 mice received 4 injections of recMASH2+AS15 or AS15 alone before challenge with TC1-MASH2 tumor cells (Tumor Challenge). *Apc*^*+/Min-FCCC*^ mice received 9 injections of recMASH2+AS15 or vehicle (phosphate buffer saline [PBS] or AS15 alone), before (two independent Prophylactic Studies) or after (Immunotherapy) colon adenomas were detectable by colonoscopy. CB6F1 mice immunized with recMASH2+AS15 had a significantly smaller mean tumor size and improved survival rate compared to controls (104 mm^2^ vs. 197 mm^2^ [p = 0.009] and 67% vs. 7% [p = 0.001], respectively). In Prophylactic Study 1, the mean number of colon adenomas was significantly lower in *Apc*^*+/Min-FCCC*^ mice receiving recMASH2+AS15 compared to PBS (1.8 [95% confidence interval 1.0–3.3] vs. 5.2 [3.7–7.4], p = 0.003). Fewer microadenomas were observed in recMASH2+AS15 groups compared to PBS in both Prophylactic Studies (Study 1: mean 0.4 [0.2–1.0] vs. 1.5 [0.9–2.4], p = 0.009; Study 2: 0.4 [0.2–0.6] vs. 1.1 [0.8–1.5], p = 0.001). In the Immunotherapy Study, fewer colon adenomas tended to be observed in recMASH2+AS15-treated mice (4.1 [2.9–6.0]) compared to controls (AS15 4.7 [3.3–6.6]; PBS 4.9 [3.5–6.9]; no significant difference). recMASH2+AS15 induced MASH2-specific antibody and CD4+ responses in both mouse models. recMASH2+AS15 partially protected mice against MASH2-expressing tumors and reduced spontaneous colorectal adenomas in *Apc*^*+/Min-FCCC*^ mice, indicating that MASH2/HASH2 antigens are targets for colorectal cancer immunotherapy.

## Introduction

Colorectal cancer (CRC) is one of the most common cancers of the Western world and a leading cause of cancer-related mortality [1–3]. Unfortunately, 30%–40% of CRC patients have local, regionally advanced or metastatic disease that cannot be cured by surgery [4]. Despite recent progress in diagnosis and treatment, the prognosis of patients with advanced CRC remains poor [5].

Genetic and environmental factors contribute to the risk of developing CRC [6–12]. Primary prevention efforts continue to focus on either reducing factors that confer CRC risk or intervening with chemopreventive agents. Populations at highest risk for CRC (i.e. individuals >age 50, with a family history of CRC or inflammatory bowel disease) continue to be the target of screening programs that utilize various molecular techniques to detect malignancy at an early stage [6, 8, 10, 13–15]. While several agents have been identified that can prevent or suppress the progression of precursor lesions, adverse effects occur. The chemopreventive activity of cyclooxygenase-2 inhibitors and aspirin is accompanied by an increased risk of cardiovascular events, and gastrointestinal and intracranial bleeding, respectively [16–21]. Thus, the search for safe and cost-effective drugs for the prevention and treatment of CRC continues.

Emerging data highlight the importance of the host immune system in controlling the growth and evolution of CRC. A complex interaction between tumor cells and the local immune response results in a balance between tumor-promoting and -controlling effects, and a close interaction of the innate and adaptive immune systems [4, 22]. In CRC patients, tumor-infiltrating immune cells were independent prognostic factors of overall and progression-free survival. Increased infiltration of CRC tumors by cytotoxic memory T-lymphocytes (i.e. CD8+ or CD45RO+) was highly correlated with reduced risk of recurrence and improved survival [23–26]. These findings suggest that mobilizing the immune system of CRC patients could lead to clinical benefit.

Various immunotherapeutic approaches have been developed to harness the immune system in combating CRC. However, despite promising results with immune checkpoint inhibitors, viral vector-based immunotherapies, dendritic cell or peptide vaccines, or irradiated autologous tumor cells, there are no approved antigen-specific cancer immunotherapies for the treatment of CRC [27, 28].

The canonical Wnt signaling pathway is involved in the renewal and proliferation of stem cells, as well as cell differentiation [29, 30]. In the absence of Wnt signaling, the adenomatous polyposis coli (APC) protein forms a complex with the GSK3β, Axin, Dsh and β-catenin proteins, leading to degradation of β-catenin. Upon stimulation of Wnt signaling, β-catenin accumulates in the cytoplasm and subsequently translocates to the nucleus where it activates the transcription of numerous oncogenes. Likewise, mutational inactivation of the *Apc* gene results in constitutive activation of Wnt signaling, continuous expression of oncogenes, and ultimately tumorigenesis [30, 31]. *Apc* mutations are present in over 80% of sporadic CRCs in humans [30–32].

The Multiple Intestinal Neoplasia (*Min*) mouse model contains a mutant allele of the *Apc* gene. Like humans with germline mutations in *Apc*, *Apc*^*+/Min*^ mice are predisposed to intestinal adenomas, but develop predominantly small intestinal adenomas and few, if any, colorectal adenomas. A unique strain of mice at Fox Chase Cancer Center (FCCC) (*Apc*^*+/Min-FCCC*^ mice) that spontaneously develops colorectal adenomas at a high multiplicity and high incidence (81.8%), as compared to the conventional *Min* mouse strain, provides a relevant model for studying colorectal neoplasia; in addition, the extended life-span of the *Apc*^*+/Min-FCCC*^ mice provides a sufficient window of opportunity for therapeutic intervention [33].

Human achaete scute homolog 2 (*Hash2* or *achaete scute-like 2*) and its murine ortholog *Mash2* encode basic helix-loop-helix transcription factors that are essential for differentiation of the mammalian trophoblast lineage [34]; HASH2 and MASH2 share 78.5% amino acid sequence homology and act as master regulators of intestinal stem cell identity [35–37]. Wnt-mediated regulation of *Hash2* expression may explain why the majority of human CRCs are HASH2-positive, as assessed by *in situ* hybridization or reverse-transcription polymerase chain reaction (52%–71%) [35, 38] and immunohistochemistry (unpublished GSK data). MASH2 is also overexpressed in intestinal adenomas that spontaneously develop in *Apc*^*+/Min-FCCC*^ mice (unpublished GSK data). Thus, HASH2 represents a potential target for CRC immunotherapy.

The purpose of the present studies was to assess the immune response induced by recombinant MASH2 protein formulated with the proprietary GSK AS15 immunostimulant (recMASH2+AS15) and evaluate its ability to control the growth of MASH2-expressing transplantable tumors in CB6F1 mice and spontaneous colon adenomas in *Apc*^*+/Min-FCCC*^ mice.

## Materials and methods

### Animal source

Female CB6F1 mice (C57BL/6 x BALB/c, 6–8 weeks of age) (transplanted tumor model) were purchased from Charles River Laboratories (St-Constant, Quebec, Canada) and allowed to acclimate for a minimum of 5 days. Male *Apc*^*+/Min-FCCC*^ mice (spontaneous tumor model) were obtained from an established breeding colony at FCCC and maintained on autoclavable Teklad 2018SX chow (Envigo, Madison, WI, USA) for the duration of the study. All animals had free access to food and water and were housed (5/cage) at 20–21°C (+/- 2 degrees) and 30–70% relative humidity, with 12 hr light/dark cycles.

### Vaccine

recMASH2+AS15 consists of purified recombinant MASH2 protein (recMASH2) produced in *Escherichia coli* (see **S1 Appendix**) and AS15, an immunostimulant containing 3-O-desacyl-4’-monophosphoryl lipid A (50 μg, produced by GSK), *Quillaja saponaria Molina*, fraction 21 (50 μg, licensed by GSK from Antigenics LLC, a wholly owned subsidiary of Agenus Inc., Delaware, USA), CpG 7909 synthetic oligodeoxynucleotides containing unmethylated CpG motifs (420 μg), and liposome.

### Study design

#### Transplanted TC1-MASH2 tumor model

**Fig 1:**
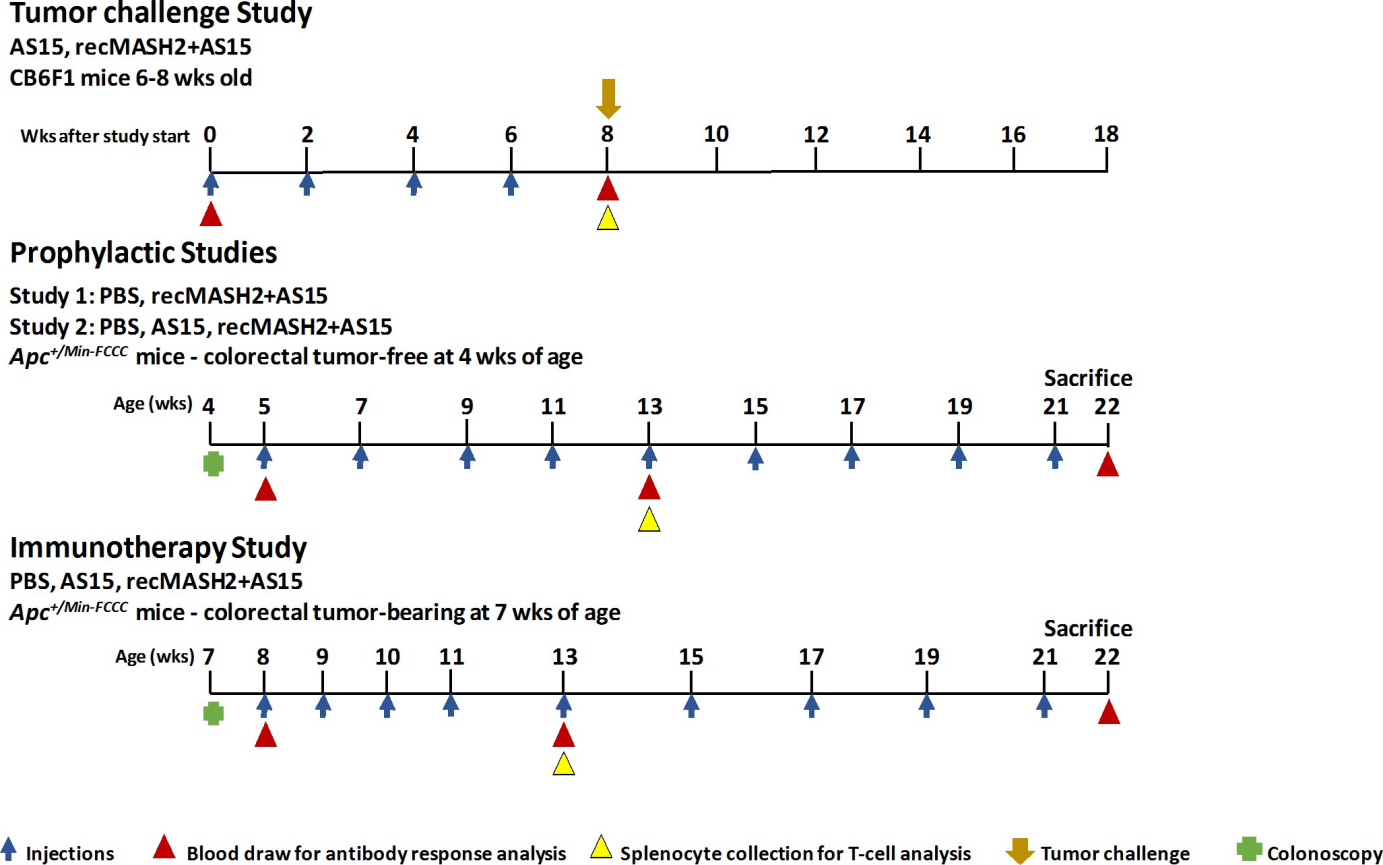
Study design. recMASH2+AS15, recombinant mouse achaete scute homolog 2 protein combined with AS15 immunostimulant; wks, weeks; PBS, phosphate buffer saline; *Apc*^*+/Min-FCCC*^, Multiple Intestinal Neoplasia mouse strain with a mutant allele of the *Apc* gene, bred at Fox Chase Cancer Center.

Female CB6F1 mice (C57BL/6 x BALB/c, 6–8 weeks of age) were maintained under pathogen-free conditions. Mice received 4 intramuscular (IM) injections (50 μl) of either 10 μg recMASH2+AS15 or AS15 alone at 2-week intervals; AS15 was used at 1/10^th^ of the human dose (**Fig 1**). Two weeks after the last immunization, mice were challenged with a subcutaneous injection of 500,000 TC1-MASH2 tumor cells (200 μl in the flank) expressing the MASH2 protein (see **S1 Appendix**). Individual tumor growth (product of the two largest diameters) was recorded 3 times a week, starting 7 days after challenge. Clinical examination was performed on a daily basis. Mice were euthanized when the tumor size reached 289 mm^2^ or at 70 days post-challenge, using 20% sodium pentobarbital solution (100 μl/mouse).

All treatments were administered in the semi-tendinous muscle of the hind leg. The tumor status of each *Apc*^*+/Min-FCCC*^ mouse was determined by colonoscopy prior to study entry. Blood samples were collected prior to treatment initiation, at week 8 in the Tumor Challenge Study (CB6F1 mice) and at 13 and 22 weeks of age in the Prophylactic and Immunotherapy Studies *(Apc*^*+/Min-FCCC*^ mice). Splenocytes were collected in week 8 for CB6F1 mice and week 13 for *Apc*^*+/Min-FCCC*^ mice.

#### Spontaneous colon tumorigenesis model

Mice were genotyped for a mutation in codon 850 of the *Apc* gene [33]. Prior to the study, the distal colon of each mouse was examined endoscopically [39] to identify tumor-free and tumor-bearing mice. Images were recorded with a QImaging Micropublisher 3.3 digital camera (QImaging Corp, Surrey, British Columbia, Canada). Colonoscopy detected lesions ≥0.5 mm in diameter within the most distal 3 cm of the colon.

In *Apc*^*+/Min-FCCC*^ mice, AS15 was used at 1/20^th^ of the human dose. All mice were monitored daily for swelling/redness at the injection site, dehydration, and cachexia. Body weights were measured at the time of injection and for 4 subsequent days. Mice exhibiting >20% weight loss were euthanized immediately by inhalation of carbon dioxide and the organs collected for analysis. All *Apc*^*+/Min-FCCC*^ mice were euthanized at study week 22 and necropsied.

For the Prophylactic Studies, *Apc*^*+/Min-FCCC*^ mice confirmed to be tumor-free by colonoscopy at 4 weeks of age were randomized one week later to receive 9 IM injections (50 μl) of either phosphate-buffered saline (PBS) or 10 μg recMASH2+AS15 (Study 1) or PBS, AS15 or 10 μg recMASH2+AS15 (Study 2) every other week (**Fig 1**). In the Immunotherapy Study, *Apc*^*+/Min-FCCC*^ mice confirmed by colonoscopy to have tumors at 7 weeks of age were randomized to receive 9 IM injections of PBS, AS15 or recMASH2+AS15. The first 4 injections were administered weekly, starting at 8 weeks of age, and the last 5 injections were given every 2 weeks (**Fig 1**).

### Histopathology

The following organs were collected from all animals for evaluation: cerebellum, trachea, salivary gland, stomach, esophagus, bladder, colon, small intestine, testis, prostate, thymus, spleen, pancreas, kidney, liver, lung, brain, skin, and heart. After formalin fixation, paraffin sections (7–10 μm thick) were stained with hematoxylin-eosin. Masson’s trichrome method, Cresyl violet or the Klüver-Barrera method was used to stain the brain samples.

At sacrifice, the entire colon and small intestine of *Apc*^*+/Min-FCCC*^ mice were excised and the number of gross colon and small intestinal tumors was counted. All tissues were fixed in 10% normal buffered formalin overnight and embedded in paraffin. Following fixation, the small intestine and colon were cut into proximal, mid and distal segments and either “jelly rolled” or cut at 2-mm intervals for paraffin embedding, respectively.

**Fig 2:**
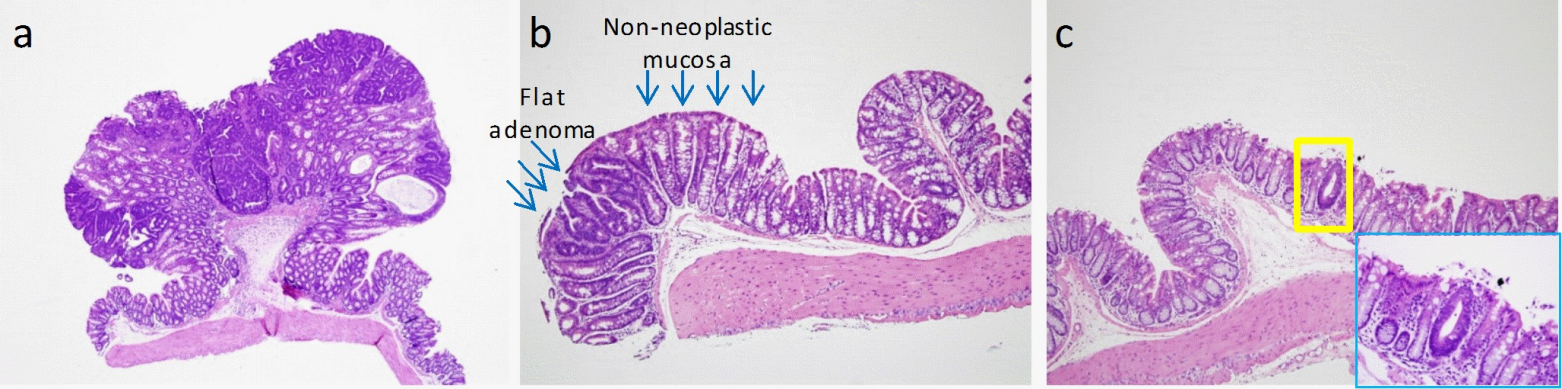
Types of colon adenomas in Apc^+/Min-FCCC^ mice. a) Polypoid adenoma (4X). Lesion protrudes above the luminal surface as compared to a flat adenoma. b) Flat adenoma (10X). Lesion is flat in nature, non-protruding, with a height that is not more than twice that of the adjacent non-neoplastic colonic mucosa. c) Microadenoma (10X). Single abnormal crypt (yellow box). High power view (40X) of the same single crypt (blue box).

Hematoxylin-eosin-stained sections were pathologically examined in a blinded manner. Adenomas were classified as polypoid, nonpolypoid, indeterminate or microadenomas (**Fig 2**). Any adenoma exhibiting an elevated growth pattern, grossly or microscopically, was considered polypoid. Nonpolypoid (flat) lesions displayed a height less than twice that of the adjacent nonneoplastic mucosa. Indeterminate adenomas did not fit into the criteria of either polypoid or flat. Microadenomas (1–4 crypts) were undetectable upon gross examination. The total number of adenomas per mouse was determined by summing the polypoid, flat, indeterminate and microadenomas. Small intestinal adenomas were identified grossly and histopathologically confirmed in cross sections of jelly rolls.

### Immunological analyses

The MASH2-specific antibody response was evaluated in sera from each mouse using an enzyme-linked immunosorbent assay. Sera were analyzed in all mice 14 days after the 4th injection (**Fig 1**) and again in *Apc*^*+/Min-FCCC*^ mice after the 9th injection. Antibody titers were determined with a mouse immunoglobulin G (IgG) standard curve using a specific capture IgG antibody.

The MASH2-specific T-cell response was evaluated by intracellular cytokine staining and flow cytometry to assess the percentage of CD4+ or CD8+ T-cells producing interferon-gamma (IFN-γ) and tumor necrosis factor-alpha (TNF-α) in splenocytes of individual mice (see **S1 Appendix**). Splenocytes were isolated 14 days after the 4^th^ (CB6F1 and *Apc*^*+/Min-FCCC*^ mice) and 9^th^ (*Apc*^*+/Min-FCCC*^ mice only) injection (**Fig 1**) and re-stimulated with a MASH2 peptide matrix (pools of 15mer peptides overlapping by 11 amino acids covering the entire MASH2 sequence) or culture media (see **S1 Appendix**).

### Statistical analyses

All analyses were performed using SAS version 9.2.

#### Tumor size in CB6F1 mice

Mean size of the tumor, calculated with a 95% confidence interval (CI), was compared between two groups using an analysis of variance model. When a mouse died prior to the end of the study (70 days post-challenge), the last measure of its tumor size was used to calculate the mean tumor size at the end of the study.

#### Tumor analyses in Apc^+/Min-FCCC^ mice

Polypoid, flat, microadenoma, and indeterminate adenomas were considered for analysis. The sum of the number of tumors in each mouse was computed. Microadenomas were further analyzed with respect to their location (proximal, mid, and distal colon). The number of tumors by type was modeled in each study using Poisson distribution. The overdispersion parameter was estimated using the deviance method and the rate of event was reported over 22 weeks (length of each study). The same approach was used to estimate the event rate in the PBS and recMASH2+AS15 groups during Prophylactic Studies 1 and 2, with study as a factor. The differences between two groups were considered significant for p-values <0.05; no pre-specified cut-off for significance was defined in the protocol.

#### Time-to-euthanasia or death

Euthanasia or death that did not occur during anesthesia was considered a ‘treatment failure’. Mice that died under anesthesia were censored at the time of death. Time-to-treatment failure was compared between groups using a Cox-proportional hazard model that included study as a factor for comparisons between the recMASH2+AS15 and control (PBS or AS15) groups. Kaplan-Meier estimates of survival are provided.

### Ethics statement

All studies with the transplantable tumor model were conducted at the Institut National de la Recherche Scientifique-Institut Armand-Frappier, Laval, Canada in accordance with the Canadian Council for Animal Care and the GSK Vaccines Policy on the Care, Welfare and Treatment of Animals. Experiments in the spontaneous colon tumor model were conducted at FCCC; Philadelphia, Pennsylvania, USA and were approved by the Institutional Animal Care and Use Committee. (Protocol Number 10–3). Both animal facilities are accredited by the Association for Assessment and Accreditation of Laboratory Animal Care. All efforts were made to minimize suffering of animals.

## Results

### recMASH2+AS15 induces immune responses and tumor protection in transplanted TC1-MASH2- tumors in CB6F1 mice

Fourteen days after the 4^th^ injection, high MASH2-specific IgG antibody levels were measured in CB6F1 mice injected with recMASH2+AS15 (geometric mean titer [GMT] of 2.54 x 10^6^ ng/ml); no response was measured in mice injected with AS15 alone. Immune response measured in spleens from the same recMASH2+AS15-immunized mice revealed a high percentage (mean of 1%) of MASH2-specific CD4+ T-cells producing cytokines (IFN-γ/TNF-α) after *in vitro* stimulation with MASH2 peptide pools (**S1 Fig**). According to the MASH2 peptide matrix (**Table A and Table B in S1 Appendix**), the immunodominant MASH2 peptides were peptide 41 (RSAVEYIRALQRLLA, 100% conserved in HASH2) and peptide 25 (PELLRCSRRRRSGAT); these peptides also contain HASH2 immunodominant CD4 epitopes identified in CB6F1 mice injected with recHASH2+AS15 (unpublished GSK data). No MASH2-specific CD8+ T-cells were observed.

**Fig 3:**
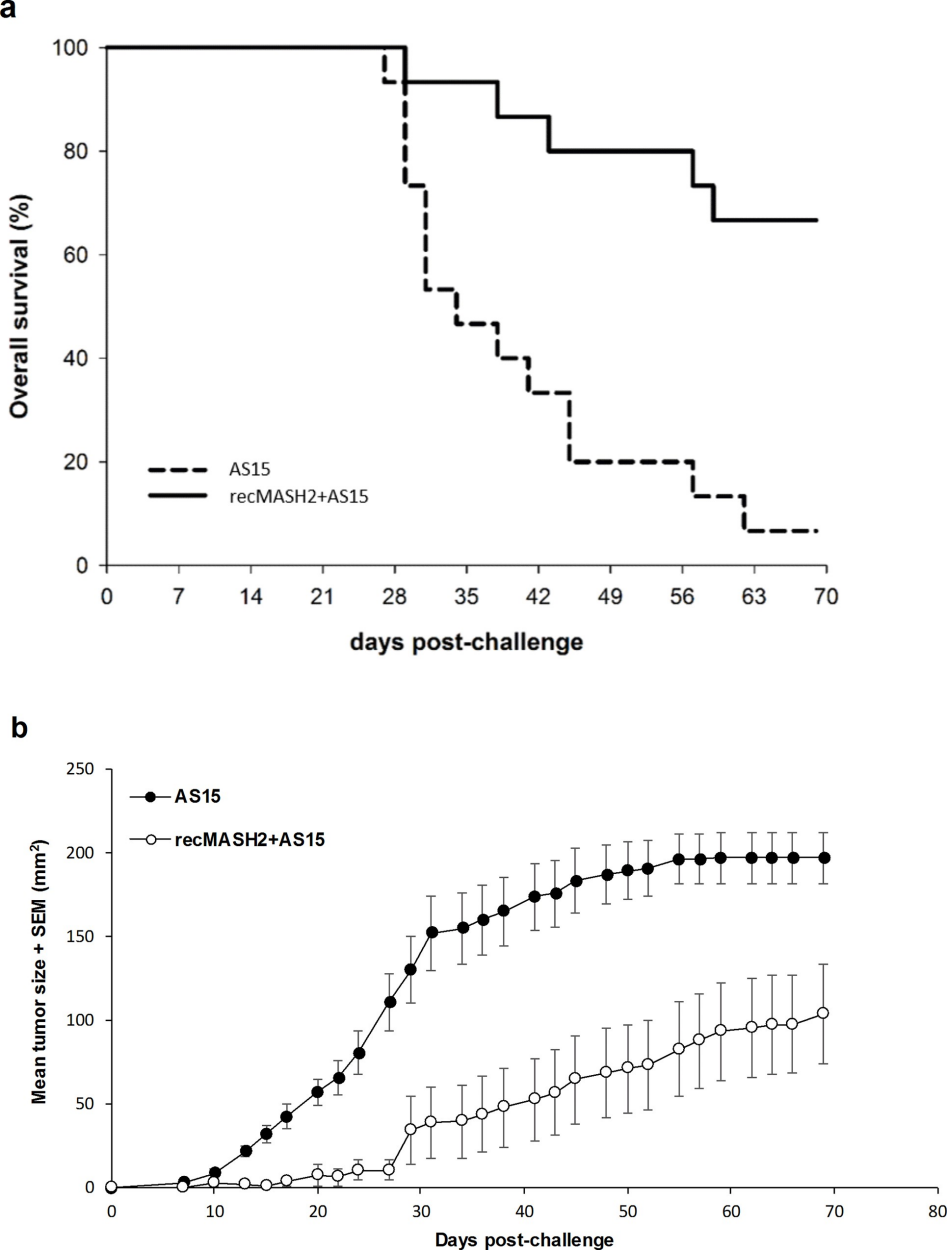
Response of CB6F1 mice to a challenge with TC1-MASH2 tumor cells. **a) Kaplan-Meier survival analysis. b) Change in tumor growth over time as determined by caliper measurements (p≤0.009).** recMASH2+AS15, recombinant mouse achaete scute homolog 2 protein combined with AS15 immunostimulant; SEM, standard error of the mean.

The antitumor activity of recMASH2+AS15 was assessed by challenging mice (15/group) with TC1-MASH2 cells and monitoring tumor growth for 70 days. Only 1 of 15 mice (7%) receiving AS15 alone survived until the end of the study and was tumor-free. In contrast, 10 of 15 mice (67%) injected with recMASH2+AS15 completed the entire study (p = 0.001), of which 7 (70%) were tumor-free (**Fig 3A**). The mean size of tumors in mice treated with recMASH2+AS15 was smaller than that of the AS15 group (**Fig 3B**). Significant differences in tumor size were observed starting on day 13 of the challenge (p<0.001) and remained significant until the end of study (104 mm^2^ for recMASH2+AS15 vs. 197 mm^2^ for AS15, p = 0.009).

### recMASH2+AS15 induces immune responses and inhibits the formation of colon and small intestinal tumors in Apc^+/Min-FCCC^ mice

To evaluate the capacity of repeat immunizations with recMASH2+AS15 to inhibit sporadic colorectal adenomas, two independent Prophylactic Studies were conducted. In both studies, male *Apc*^*+/Min-FCCC*^ mice were tumor-free at baseline. In Study 1, mice received recMASH2+AS15 (n = 20), or PBS as control (n = 25). In Study 2, mice received recMASH2+AS15 (n = 53), PBS (n = 51) or AS15 alone (n = 57). After 4 injections, a strong MASH2-specific IgG antibody response was observed in *Apc*^*+/Min-FCCC*^ mice immunized with recMASH2+AS15 (Study 1: GMT 8.9 x 10^5^ ng/ml; Study 2: 7.9 x 10^5^ ng/ml), which remained high following 9 injections (Study 1: 8.5 x 10^5^ ng/ml; Study 2: 1.1 x 10^6^ ng/ml). No response was measured in mice treated with PBS or AS15 alone. After 9 injections of recMASH2+AS15, a strong induction of MASH2-specific CD4+ T-cells producing TNF-α and IFN-γ was observed (Study 1: 9.1%; Study 2: 11.4%); no MASH2-specific CD8+ T-cells were observed. Cytokine-producing CD4+ T-cells were not induced in mice injected with either PBS or AS15 alone.

**Fig 4:**
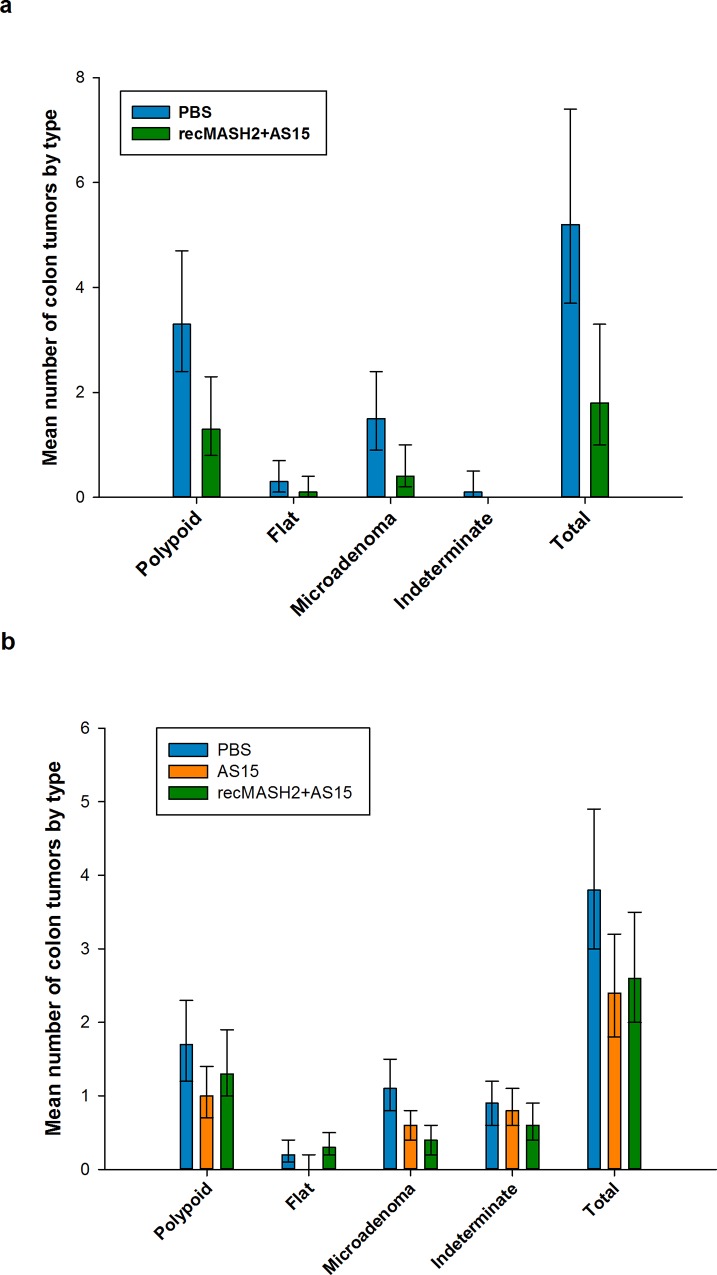
Multiplicity of colon adenomas post-treatment by morphological subtype. **Values based on histopathologically confirmed adenomas in Apc^+/Min-FCCC^ mice. a) Prophylactic Study 1 and b) Prophylactic Study 2 at necropsy.** PBS, phosphate buffer saline; recMASH2+AS15, recombinant mouse achaete scute homolog 2 protein combined with AS15 immunostimulant. Error bars represent 95% confidence intervals.

In Study 1, the total number of colon adenomas was significantly lower in the recMASH2+AS15 group versus the PBS group (mean 1.8 [95% CI 1.0–3.3] vs. 5.2 [3.7–7.4], p = 0.003) (**Fig 4A**). Fewer colon microadenomas and polypoid adenomas were observed in mice treated with recMASH2+AS15 than in the PBS group (microadenomas: mean 0.4 [0.2–1.0] vs. 1.5 [0.9–2.4], p = 0.009; polypoid adenomas: 1.3 [0.8–2.3] vs. 3.3 [2.4–4.7], p = 0.006) (**Fig 4A**). In Study 2, the antitumor activity of recMASH2+AS15 was restricted to colon microadenomas, with the mean number in recMASH2+AS15 mice significantly lower than those administered PBS (0.4 [0.2–0.6] vs. 1.1 [0.8–1.5], p = 0.001), but not AS15 (0.6 [0.4–0.8], p = 0.281) (**Fig 4B**). The mean number of flat and indeterminate adenomas did not differ significantly across the treatment groups in either Study 1 or 2 (**Fig 4A and 4B**).

**Fig 5:**
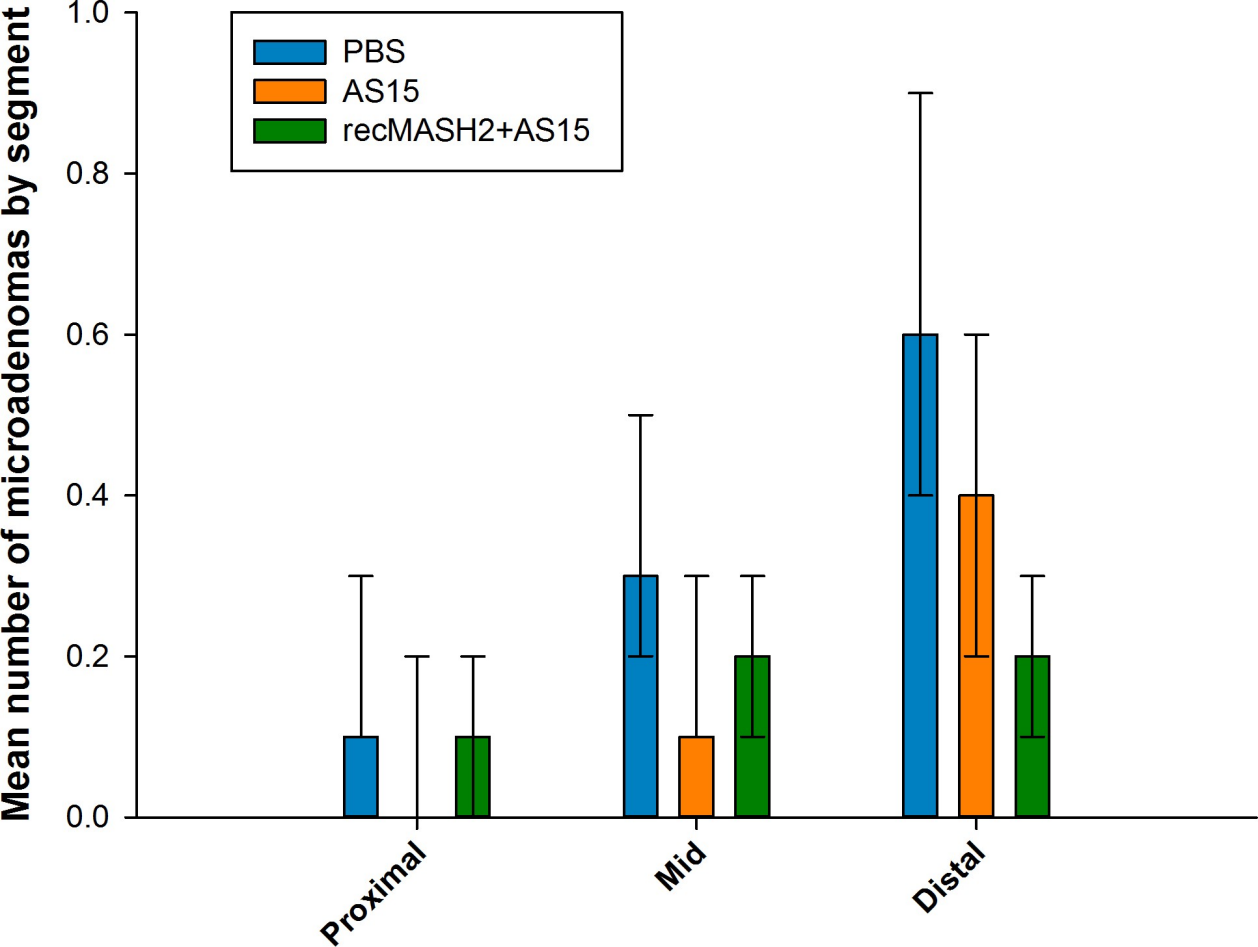
Multiplicity of microadenomas per colon segment. PBS, phosphate buffer saline; recMASH2+AS15, recombinant mouse achaete scute homolog 2 protein combined with AS15 immunostimulant. Values are based on histopathological reviews of mice in Prophylactic Study 2 at necropsy. Error bars represent 95% confidence intervals.

The same trend was observed when data from all mice in both studies were pooled, with a significantly lower number of total microadenomas in the recMASH2+AS15 group vs. PBS (0.4 vs. 1.2, p<0.001). With respect to location, the largest response to recMASH2+AS15 treatment in Study 2 was observed among distal microadenomas (mean 0.2 [0.1–0.3] vs. 0.6 [0.4–0.9] in the PBS group, p<0.001, and 0.4 [0.2–0.6] in the AS15 group, p = 0.032; **Fig 5**).

The ability of recMASH2+AS15 to inhibit the formation of colorectal tumors was also evident from an increased number of tumor-free mice at the end of the study. The majority (74%) of the gross colon adenomas identified at necropsy were located within the region accessible to the endoscope. In Study 1, 4/19 (21%) mice in the recMASH2+AS15 group were tumor-free at necropsy, as compared to 2/20 (10%) of PBS-injected controls. Similar results were obtained in Study 2, where the number of *Apc*^*+/Min-FCCC*^ mice without colon tumors was higher following administration of recMASH2+AS15 (11/47; 23%) than AS15 alone (8/52;15%) or PBS (5/46; 11%).

Although the objective of the present study was to assess the impact of repeat injections of recMASH2+AS15 on the development of colon tumors, administration of recMASH2+AS15 also significantly inhibited the formation of spontaneous small intestinal adenomas in *Apc*^*+/Min-FCCC*^ mice. In Study 1, the mean number of small intestinal adenomas was significantly lower in the recMASH2+AS15 group vs. the PBS group (9.2 [6.3–13.6] vs. 17.5 [13.2–23.2], p = 0.011; **S2 Fig**). This reduction was observed in the proximal, mid and distal small intestine. In Study 2, the mean number of small intestinal adenomas was also significantly lower in the recMASH2+AS15 group compared to the PBS group (14.4 [11.9–17.4] vs. 22.7 [19.5–26.6], p<0.001); no significant difference between the recMASH2+AS15 and AS15 groups was detected (p = 0.133). This decrease in tumor number was observed primarily in the mid and distal small intestine (**S2 Fig**).

**Fig 6:**
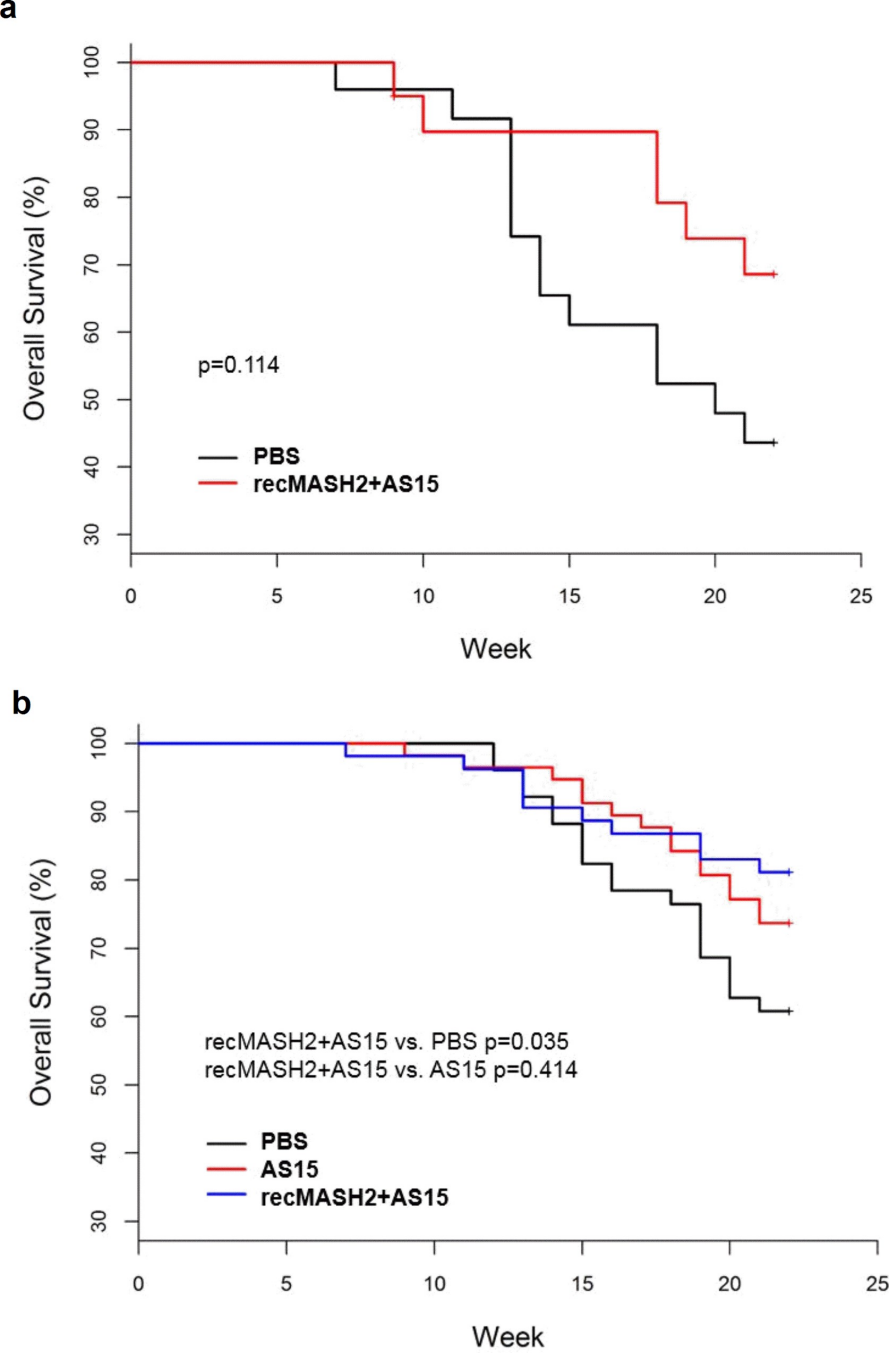
**Overall survival of Apc^+/Min-FCCC^ mice in a) Prophylactic Study 1 and b) Prophylactic Study 2.** PBS, phosphate buffer saline; recMASH2+AS15, recombinant mouse achaete scute homolog 2 protein combined with AS15 immunostimulant.

Despite developing a more severe colon phenotype, *Apc*^*+/Min-FCCC*^ mice live longer (~150 days) than conventional *Apc*^*+/Min*^ mice and can be treated until 22 weeks of age. Treatment of *Apc*^*+/Min-FCCC*^ mice with recMASH2+AS15 led to a 22% absolute increase in the number of mice that completed the entire regimen in Study 1 (22 weeks) as compared to the PBS control group (p = 0.114). A similar trend was observed in Study 2, where a higher number of mice completed the 22-week study regimen in the recMASH2+AS15 group compared to the PBS group (81% vs. 61%, p = 0.035); although not statistically significant when compared to AS15 alone (74% mice finished the study, p = 0.4139). The overall survival for the *Apc*^*+/Min-FCCC*^ mice is presented in **Fig 6**. When pooling the data from Study 1 and 2, a significant increase in the number of mice surviving until the end of study was observed in the recMASH2+AS15 group vs. the PBS group (p = 0.008).

### Immunogenicity and antitumor activity of recMASH2+AS15 in tumor-bearing Apc^+/Min-FCCC^ mice

To evaluate the capacity of repeat immunizations with recMASH2+AS15 to alter the growth of established tumors, male *Apc*^*+/Min-FCCC*^ mice with endoscopically-confirmed tumors received 9 injections of PBS, AS15, or recMASH2+AS15. Similar to the Prophylactic Studies, male *Apc*^*+/Min-FCCC*^ mice immunized with recMASH2+AS15 showed strong MASH2-specific immune responses with high levels of IgG antibodies (GMT approximately 1 x 10^6^ ng/ml after 4 and 9 immunizations) and CD4+ T-cells producing TNF-α and IFN-ϒ (10%), but no CD8+ T-cells were detected. Although the mean multiplicity of colon adenomas was lowest at the end of the study in mice treated with recMASH2+AS15 (4.1 [2.9–6.0]) compared to controls (AS15 4.7 [3.3–6.6]; PBS 4.9 [3.5–6.9]), none of the comparisons reached statistical significance (**S3 Fig**). The largest difference in mean multiplicity between control and treated groups was observed among microadenomas (mean 0.8 [0.4–1.6], 1.3 [0.8–2.2], and 1.4 [0.8–2.3] in the recMASH2+AS15, PBS, and AS15 groups, respectively; no significant differences). Similar to Prophylactic Study 2, the difference between groups in the number of microadenomas per animal was most pronounced in the distal region (mean of 0.4 [0.2–0.8], 0.7 [0.4–1.3], and 0.6 [0.3–1.2], in the recMASH2+AS15, PBS, and AS15 groups, respectively; no significant differences) (**S3B Fig**).

### Evaluation of safety

Because low levels of MASH2 mRNA are expressed in select normal murine tissues [34, 40, 41], potential treatment-induced toxicity was assessed. Similar to C57Bl/6 control mice, very low levels of MASH2 mRNA were detected in the normal colon and small intestine of *Apc*^*+/Min-FCCC*^ mice. However, MASH2 mRNA was overexpressed in spontaneous adenomas of the colon and small intestine (unpublished GSK data). No histological abnormalities indicative of organ toxicity were observed in the bladder, brain, cerebellum, esophagus, heart, kidneys, liver, pancreas, prostate, salivary glands, skin, spleen, testes, thymus or trachea of recMASH2+AS15-treated CB6F1 (4 injections) or *Apc*^*+/Min-FCCC*^ (9 injections) mice, or in the colon and small intestine of recMASH2+AS15-treated CB6F1 mice. Body weights of *Apc*^*+/Min-FCCC*^ mice were comparable between the control group and study groups (data not shown). No significant loss (> 20%) was observed during the experiments. Pulmonary inflammation was observed in *Apc*^*+/Min-FCCC*^ mice, irrespective of the study group and was unrelated to treatment.

## Discussion and conclusions

The results of our studies indicate the capacity of the recombinant MASH2 protein (a self-antigen for mice), formulated with AS15 immunostimulant, to induce humoral and cellular immune responses. recMASH2+AS15 significantly decreased the growth of MASH2-expressing transplanted tumors in CB6F1 mice, reduced the number of spontaneous colon adenomas in *Apc*^*+/Min-FCCC*^ mice and increased the overall survival of mice in both models.

Although transplantable tumors are easy to handle in a laboratory setting, they grow fast and may not fully mimic the slow tumor growth in human patients. Therefore, we have also evaluated the impact of recMASH2+AS15 immunotherapy on the spontaneous development of intestinal tumors in *Apc*^*+/Min-FCCC*^ mice, an animal model, which more closely reflects the way CRC develops in its natural site, and in which the results are expected to be more predictive of therapeutic outcome in future human trials. Experiments were designed to mimic either prophylaxis in patients at high risk for CRC or its recurrence, or immunotherapy in patients with existing colon tumors. In both experiments, recMASH2+AS15 reduced the formation of colorectal microadenomas.

Injection of mice with recMASH2+AS15 resulted in strong induction of MASH2-specific antibodies and cytokine-producing CD4+ T-cells. No CD8+ T-cell response was detected, consistent with previous data from mice injected with AS15 combined with another recombinant protein coding for another tumor antigen, MAGE-A3 [42]. As demonstrated in CD8+ T-cell-depleted mice or perforin-KO mice, CD8+ T-cells did not account for the antitumor response observed in a MAGE-A3-transfected tumor model and CD4+ T-cells were the critical effector. Since antitumor activity was observed in the absence of CD8+ T-cell responses in the present study, CD4+ T-cells seem to be the critical effector in CRC as well. MASH2 is an intracellular protein; thus, antibodies are not expected to play a direct role in tumor cell killing through antibody-dependent cellular cytotoxicity. However, immune complexes can be taken up by antigen presenting cells via their Fcγ receptor and cross-presented to T-cells, thereby indirectly contributing to tumor rejection [43, 44]. Because cytotoxic CD8+ T-cells are often considered the primary cells responsible for tumor elimination in humans [23, 26, 45, 46], the addition of an approach to induce CD8+ T-cell response could lead to improved HASH2 efficacy in humans. A more potent immunostimulant, including recombinant adenoviruses or RNA vaccines, could be a good candidate for this purpose [28]. However, induction of CD4+ T-cell responses would be a prerequisite, as they accompany the observed antitumor activity of MASH2 and help induce and maintain CD8+ T-cell responses [47–49].

recMASH2 coupled with the AS15 immunostimulant was immunogenic and increased the disease-free survival of *Apc*^*+/Min-FCCC*^ mice. However, this strategy alone may be insufficient to provide clinical benefit to cancer patients, requiring combination therapy. One option would be use in combination with cyclophosphamide to break regulatory T-cell-induced tolerance to immunotherapy [50]. Monoclonal antibodies that block immune checkpoints are extremely efficacious in inducing clinical responses in metastatic melanoma and other solid tumors [51, 52]. Such treatment used in combination with antigen-specific immunotherapy may help sustain the immune response and improve clinical benefit. Viral vector approaches, as well as standard chemotherapy or radiotherapy, could also be considered [28].

The concept of HASH2-based immunotherapy for CRC is based on several observations. First, expression of HASH2 in the non-neoplastic intestine is restricted to cells at the base of the crypt, where the protein plays a critical role in controlling stem cell fate [35]. Transgenic expression of HASH2 in the intestinal epithelium leads to crypt hyperplasia, while induced deletion in the adult small intestine causes rapid disappearance of Lgr5-positive progenitors [53]. Second, HASH2 is overexpressed in the majority (up to 71%) of human CRC, with levels elevated 15-fold [35, 37, 38, 53]. By immunohistochemistry using a specific anti-HASH2 monoclonal antibody, we also confirmed the overexpression of HASH2 protein in human colorectal tumors and metastases, compared to adjacent normal tissues (unpublished GSK data). Third, and most importantly, β-catenin-mediated TCF signaling, the target of HASH2, is one of the earliest aberrations in colorectal carcinogenesis. Inhibition of this “gatekeeping” oncogenic event is consistent with the ability of recMASH2+AS15 to dramatically decrease the multiplicity of microadenomas following prophylactic and immunotherapeutic treatments. In humans, microadenomas are the earliest neoplastic colon lesions that are detected histologically, precursors of “classic” adenomas. Lastly, deregulation of the Wnt pathway leading to methylation of HASH2 is a prognostic factor for CRC recurrence after therapy [54]. Methylation and overexpression of HASH2 has been correlated with poor prognosis and resistance to chemotherapy in gastric cancer [55]. Together, these data highlight the homology between HASH2 and MASH2, and indicate that HASH2 represents a promising target for early immunopreventive intervention in colon carcinogenesis.

Although the HASH2 antigen and its ortholog MASH2 are not only localized to CRC cells [35, 37, 56], and also present in Lgr5-positive colon progenitors [53, 57], no safety concerns were identified in this study. Transitory expression of HASH2 or MASH2 mRNA and protein in normal tissues, upon Wnt activation, may explain the absence of adverse events after vaccination with recMASH2+AS15. As CD8+ T-cell responses were not induced, a more potent immunostimulant could be considered in the future, providing an acceptable safety profile can be obtained.

In conclusion, the results of the present study demonstrate that MASH2 and, by extrapolation, HASH2 could be viable targets for immunotherapy of CRC.

## Supporting information

S1 Fig Percentage of CD4+ T-cells producing cytokines (IFN-γ and/or TNF-α) in CB6F1 mice after stimulation with a matrix of 16 peptide pools covering the whole MASH2 sequence or with an irrelevant stimulation (Roswell Park Memorial Institute medium). SEM, standard error of the mean.
(TIF)

S2 Fig Multiplicity of small intestinal adenomas post-treatment. Values represent histologically confirmed adenomas in the small intestine of mice in Prophylactic Study 1 (a) and Prophylactic Study 2 (b) at necropsy and processed as jelly rolls.
PBS, phosphate buffer saline; recMASH2+AS15, recombinant mouse achaete scute homolog 2 protein combined with AS15 immunostimulant.
The error bars represent 95% confidence intervals.
(TIF)

S3 Fig Multiplicity of colon adenomas post-treatment by morphological subtype. Values represent histopathologically confirmed colon adenomas (a) and microadenomas (b) at necropsy in mice bearing colon tumors at the time of treatment initiation (Immunotherapy Study).
PBS, phosphate buffer saline; recMASH2+AS15, recombinant mouse achaete scute homolog 2 protein combined with AS15 immunostimulant.
The error bars represent 95% confidence intervals.
(TIF)

S1 Appendix This file contains supplementary methods for the production of the MASH2 vaccine and associated immunological analyses. (DOCX)
