# Supplementary material for: Self-antigen MASH2 combined with the AS15 immunostimulant induces tumor protection in colorectal cancer mouse models
Source: PLoS One. 2019 Jan 25;14(1):e0210261. doi: 10.1371/journal.pone.0210261 (PMC6347180; doi:10.1371/journal.pone.0210261)
Supplement: S1 Appendix — (DOCX) [file pone.0210261.s004.docx]

**S1 Appendix**

**Recombinant MASH2 protein production**

MASH2 (His-MASH2-w/o exon 1-w/o polyproline domain) is a purified *Escherichia coli* recombinant protein corresponding to the MASH2 sequence (263 amino acids [AA]) deleted of encoding sequences AA 1–68 (corresponding to the first exon), to generate a recombinant protein which is more similar to the human HASH2 protein, and AA 197–220 (corresponding to the poly-proline-like domain; NH2-DECAQPSASPASASLSCASTSPSP-COOH), to allow overexpression in *E. coli.* For the expression of the recombinant MASH2 protein (194 AA), a plasmid (LVL159) was designed encoding 23 AA of a hinge region in N-terminal containing 10 histidine (His) residues for purification purposes and the 171 AA of the MASH2 protein, as described above. The MASH2 cDNA was amplified on a synthetic gene, with *E. coli* optimized codons, provided by GeneArt (see the GeneArt 0709989 template below) and cloned into the pET19b vector (Novagen) using primers CAN338 and CAN278 (see the sequences below). The construct was used to transform *E. coli* strain BLR(DE3) according to standard procedures and transformed cells were selected on LB Alternative Protein Source (APS) agar plates with 100 µg/ml carbenicilin.

BLR(DE3)/LVL159 transfectant was grown in LB APS medium with 100 µg/ml carbenicilin at 37°C to reach an OD 600 of 0.5 to 1.0. Inductions were done by the addition of 1 mM IPTG and maintained at 16°C for 16h. Cultures were centrifuged (15 min, 4°C, 6000 x g) and bacterial pellets were resuspended in Lysis buffer (20 mM Tris buffer [pH 8.0] containing 500 mM NaCl, 10 mM tris(2-carboxyethyl)phosphine [TCEP]) with a mixture of protease inhibitors (complete EDTA-free). Bacteria were lyzed with the Constant Cell disruption system (Constant System) and centrifuged at 20 000 x g for 20 min at 4°C. The insoluble components (pellets) were resolubilized in 50 mM bicine buffer (pH 8.0) containing 6 M guanidine HCl, 500 mM NaCl, 10 mM TCEP, and then centrifuged (20 000 x g for 20 min). The supernatant was loaded on a 5 ml His Trap column (GE Healthcare). The 6 M GnHCl chaotrope agent was replaced by 8 M Urea (8 M Urea, 20 mM 4-(2-hydroxyethyl)-1-piperazineethanesulfonic acid [HEPES], 500 mM NaCl, 1 mM TCEP [pH 8.0]) during washing steps. Elution was performed using a 20 mM HEPES buffer (pH 8.0) containing 8 M Urea, 500 mM NaCl, 1 mM TCEP and 250 mM imidazole. One or two steps of size exclusion chromatography (Superdex 200, GE Healthcare) were done in 8 M Urea, 20 mM HEPES, 150 mM NaCl, 1 mM TCEP (pH 8.0), before the last desalting step. The desalting was done on a G25 desalting column pre-equilibrated with the final 20 mM HEPES buffer (pH 7.0) containing 4 M Urea, 150 mM NaCl and 1 mM TCEP. Protein samples were 1 ml-aliquoted in tubes and stored at -80°C.

**TC1-MASH2 cells generation**

TC-1 parental tumor cells obtained from Dr T. C. Wu (Johns Hopkins University, Baltimore, MD) were stably transfected with plasmid LVL191, constructed from pcDNA3.1/Zeocin, coding for MASH2 protein (GenBank AF139595) deleted of AA 1–68 (corresponding to the first exon, see the sequences below). Transfections were done using Fugene-6 (Roche, Mississauga, ON, Canada), according to the manufacturer’s recommendations. After transfection, cells were harvested and distributed in 96-well plates for limiting dilutions. MASH2-positive clones were then selected by addition of 100 μg/ml zeocin (Invitrogen, Burlington, ON, Canada), amplified and frozen. Total RNA from TC1-MASH2 cells was isolated using a QIAshredder homogenizer followed by DNase treatment and RNeasy purification (Qiagen, Toronto, ON, Canada).

The expression of MASH2 mRNA in TC1-MASH2 cells was verified by real-time qRT-PCR, using primers and Taqman MGB probe specific for the MASH2 sequence in plasmid LVL191:

- MASH2 LVL191 forward primer (CARA033): CTACAGCAGCGAGGAAAGCA

- MASH2 LVL191 reverse primer (CARA034): GTCCAGCAGTTCCTGTTCCATAG

- MASH2 LVL191 probe (CARA035): 6-FAM-CTGCGAGGGCGAGC-MGB-NFQ

MASH2 protein expression was verified by Western Blot using an anti-HASH2 monoclonal antibody (clone 40-12). MHC I Kb expression was evaluated by flow cytometry using the antibody anti-H2Kb FITC (Pharmingen, ref 553569 – clone AF6-88.5) and an antibody against MHC I Kd (anti-H2Kd FITC, Pharmingen, ref 553565 - clone SF1-1.1) was used as negative control (data not shown). Based on these data, the TC-1-MASH2 clone 2-9 was chosen.

To determine the optimal dose of TC1-MASH2 clone 2-9 cells for tumor challenge in mice, various doses of cells (from 1x10^5^ to 1x10^6^ cells per mouse) were injected in CB6F1 mice. The optimal dose was determined to be 500 000 TC1-MASH2 clone 2-9 tumor cells per mouse based on the generation of 289 mm^2^ (17 mm x 17 mm) tumors in approximately 30 days after challenge in CB6F1 mice.

TC1-MASH2 clone 2-9 cells were grown *in vitro* at 37°C with 5% CO_2_ in Roswell Park Memorial Institute (RPMI) medium with 10% fetal calf serum, 1% L-glutamine, 1% penicillin-streptomycin, 1% non-essential AA, 1% sodium pyruvate, and 0.1% β-mercaptoethanol. To prepare the tumor cell injections, the TC1-MASH2 clone 2-9 cells were trypsinized, washed twice in phosphate-buffered saline (PBS) and 1x10^6^ cells were re-suspended in 200 µl PBS.

**Primers used to amplify recMASH2 cDNA provided by GeneArt 0709989 for cloning into pET19b vector (Novagen)**

**CAN338** cgtcagcatatggatggtggtgcgctgccgcg

**CAN278** ggagctctcgagttaatagccgcccagccagctgc

**LVL159**

**MASH2 encoding DNA sequence in LVL159 (His-MASH2-w/o exon 1 –w/o polyproline domain):**

atgggccatcatcatcatcatcatcatcatcatcacagcagcggccatatcgacgacgacgacaagcatatggatggtggtgcgctgccgcgtctgatgccgaccagctctggtgtggcgggtgcgtgtgcggcgcgtcgtcgtcaggcgagcccggaactgctgcgctgtagccgccgtcgtcgtagcggtgcgaccgaagcgagcagcagcagcgcggcggttgcgcgtcgtaacgaacgtgaacgtaaccgtgtgaaactggtgaacctgggctttcaggcgctgcgtcagcatgtgccgcatggcggtgcgaacaaaaaactgagcaaagtggaaacgctgcgctctgcggtggaatatattcgtgcgctgcaacgcctgctggccgaacatgatgcggttcgtgcggcgctggcaggtggtctgctgacgccggcgaccccgccgagcgatcgtctgggttgcagcgaaccgaccagcccgcgtagcgcgtatagcagcgaagaaagcagctgcgaaggcgaactgagcccgatggaacaggaactgctggattttagcagctggctgggcggctattaa

**MASH2 amino acid sequence encoded by LVL159 (His-MASH2-w/o exon 1 –w/o polyproline domain)**:

MGHHHHHHHHHHSSGHIDDDDKHMDGGALPRLMPTSSGVAGACAARRRQASPELLRCSRRRRSGATEASSSSAAVARRNERERNRVKLVNLGFQALRQHVPHGGANKKLSKVETLRSAVEYIRALQRLLAEHDAVRAALAGGLLTPATPPSDRLGCSEPTSPRSAYSSEESSCEGELSPMEQELLDFSSWLGGY

**LVL191**

**MASH2 encoding DNA sequence in LVL191 (MASH2-w/o exon 1):**

atggacggaggagcactgcccaggctgatgcctaccagctctggcgtcgcaggcgcatgtgctgccagaagaaggcaggccagccccgagctgctgaggtgcagcaggcggagaagaagcggcgccaccgaggccagctctagcagcgccgctgtggccagaagaaacgagagagagaggaacagggtcaagctggtgaacctgggcttccaggccctgaggcagcacgtgccccacggcggagctaacaagaagctgtccaaggtcgagacactgagaagcgccgtggagtacatcagggccctccagaggctgctggccgagcacgacgccgtgagagccgctctcgctggcggactgctgacccccgccaccccccctagcgacgagtgcgcacagccaagcgcctctcctgccagcgcctccctgagctgcgccagcaccagccctagccccgacaggctgggctgctccgagcctaccagccccagaagcgcctacagcagcgaggaaagcagctgcgagggcgagctgtcccctatggaacaggaactgctggacttcagcagctggctgggcggctactga

**MASH2 amino acid sequence encoded by LVL191 (MASH2-w/o exon 1):**

MDGGALPRLMPTSSGVAGACAARRRQASPELLRCSRRRRSGATEASSSSAAVARRNERERNRVKLVNLGFQALRQHVPHGGANKKLSKVETLRSAVEYIRALQRLLAEHDAVRAALAGGLLTPATPPSDECAQPSASPASASLSCASTSPSPDRLGCSEPTSPRSAYSSEESSCEGELSPMEQELLDFSSWLGGY

**Geneart 0709989**

**MASH2 encoding DNA sequence in GeneArt 0709989 (MASH2 w/o polyproline domain):**

atggaagcgcatctggattggtatggcgtgccgggcctgcaagaagcgagcgacgcctgcccgcgtgaaagctgtagctctgccctgccggaagcacgtgaaggcgcgaacgtgcattttccgccgcatccggtgccgcgtgaacatttttcttgcgctgccccggaactggttgcgggtgcgcagggtctgaacgcgagcctgatggatggtggtgcgctgccgcgtctgatgccgaccagctctggtgtggcgggtgcgtgtgcggcgcgtcgtcgtcaggcgagcccggaactgctgcgctgtagccgccgtcgtcgtagcggtgcgaccgaagcgagcagcagcagcgcggcggttgcgcgtcgtaacgaacgtgaacgtaaccgtgtgaaactggtgaacctgggctttcaggcgctgcgtcagcatgtgccgcatggcggtgcgaacaaaaaactgagcaaagtggaaacgctgcgctctgcggtggaatatattcgtgcgctgcaacgcctgctggccgaacatgatgcggttcgtgcggcgctggcaggtggtctgctgacgccggcgaccccgccgagcgatcgtctgggttgcagcgaaccgaccagcccgcgtagcgcgtatagcagcgaagaaagcagctgcgaaggcgaactgagcccgatggaacaggaactgctggattttagcagctggctgggcggctatcatcatcatcaccatcattaa

**MASH2 amino acid sequence encoded by GeneArt 079989 template (MASH2 w/o polyproline domain):**

MEAHLDWYGVPGLQEASDACPRESCSSALPEAREGANVHFPPHPVPREHFSCAAPELVAGAQGLNASLMDGGALPRLMPTSSGVAGACAARRRQASPELLRCSRRRRSGATEASSSSAAVARRNERERNRVKLVNLGFQALRQHVPHGGANKKLSKVETLRSAVEYIRALQRLLAEHDAVRAALAGGLLTPATPPSDRLGCSEPTSPRSAYSSEESSCEGELSPMEQELLDFSSWLGGYHHHHHH

**T-cell responses in CB6F1 and *Apc^+/Min-FCCC^* mice**

***Re-stimulation of T-cells in vitro***

Splenocytes were isolated from injected animals and resuspended at a final concentration of 10x10^6^ cells/ml in RPMI 1640 culture medium with the following additives: penicillin-streptomycin (1X), MEM non-essential AA (1X), 2-mercaptoethanol (55 mM), sodium pyruvate (100 mM), L-Glutamine (200 mM). Live splenocytes (10^6^ cells) were re-stimulated *in vitro* for 2h at 37°C in 96-round bottom well plates with either RPMI buffer (RPMI 1640 + additives + 5% heat-inactivated Fetal Bovine Serum [FBS]) or with a bank of 63 peptides covering the entire sequence of MASH2 protein (1 µg/mL for each peptide in RPMI buffer); each peptide was a 15-mer, overlapping the next peptide by 11 AA (Table A). To investigate the immunodominant regions, 16 different pools of peptides were arranged according to a matrix such that any 2 pools of peptides had only 1 common peptide (Table B).

The re-stimulation of splenocytes was done in a final volume of 200 µl RPMI with 5% FBS containing co-stimulatory rat anti-mouse anti-CD49d and anti-CD28 antibodies (Becton Dickinson [BD]) at 2 µg/ml each. After an incubation of 2h at 37°C, the secretion of cytokines was blocked by the addition of 50 µl of brefeldin (Golgi Plug, BD; 1/1000) in RPMI with 5% FBS.

***CD4+ and CD8+ T-cell staining***

Cells were transferred to a 96-conical bottom well plate, centrifuged (300 x g for 5 min at 4°C) and washed with 200 µl Fluorescence-Activated Cell Sorting (FACS) buffer (PBS with 1% FBS). The cell pellets were incubated during 10 min at 4°C with 50 µl of rat anti-mouse CD16/CD32 antibody (2.4G2, BD; 0.5 mg/mL) in FACS buffer, protected from light, to block non-specific binding to Fcɣ receptors. CD4+ and CD8+ T-cells were stained (30 min at 4°C, protected from light) by addition of 50 µl of master mix containing fluorescent antibodies CD4 APC-Cy7mAb (1/200; BD) and CD8a PerCP-Cy5.5mAb (1/200; BD) in FACS buffer.

***Permeabilization and cytokine staining***

Cells were washed in FACS buffer and centrifuged (300 x g for 5 min at 4°C). The cells were fixed in 200 µl of cytoFix-cytoPerm solution (BD) for 20 min at 4°C and then centrifuged (500 x g for 5 min at 4°C). Cells were washed with 200 µl a 1x concentration of permeabilizing solution (perm WASH; BD) in sterile water and centrifuged (500 x g for 5 min at 4°C).

Pellets were incubated 2h at 4°C protected from light with 50 µl of a mix of fluorescent antibodies against IFN-γ APC mAb (1/150; BD) and TNF-α PE-Cy7 mAb (1/150; BD) in the perm WASH 1x solution (BD). Cells were washed, centrifuged (500 x g for 5 min at 4°C) and re-suspended in BD Stabilizing fixative (1x; BD) before FACS analysis (Canto II from BD). Data are expressed in percentages of double-positive IFN-γ^+^ and TNF-α^+^ CD4+ or CD8+ T-cells.

**Table A.** Sequences of the 63 15-mer overlapping peptides in the MASH2 matrix used for T-cell immunogenicity assessment

|  | **MASH2 peptides** |
| --- | --- |
| **1** | MEAHLDWYGVPGLQE |
| **2** | LDWYGVPGLQEASDA |
| **3** | GVPGLQEASDACPRE |
| **4** | LQEASDACPRESCSS |
| **5** | SDACPRESCSSALPE |
| **6** | PRESCSSALPEAREG |
| **7** | CSSALPEAREGANVH |
| **8** | LPEAREGANVHFPPH |
| **9** | REGANVHFPPHPVPR |
| **10** | NVHFPPHPVPREHFS |
| **11** | PPHPVPREHFSCAAPE |
| **12** | VPREHFSCAAPELVA |
| **13** | HFSCAAPELVAGAQG |
| **14** | AAPELVAGAQGLNAS |
| **15** | LVAGAQGLNASLMDG |
| **16** | AQGLNASLMDGGALPR |
| **17** | NASLMDGGALPRLMPT |
| **18** | MDGGALPRLMPTSSG |
| **19** | ALPRLMPTSSGVAGA |
| **20** | LMPTSSGVAGACAAR |
| **21** | SSGVAGACAARRRQA |
| **22** | AGACAARRRQASPEL |
| **23** | AARRRQASPELLRCS |
| **24** | RQASPELLRCSRRRR |
| **25** | PELLRCSRRRRSGAT |
| **26** | RCSRRRRSGATEASS |
| **27** | RRRSGATEASSSSAA |
| **28** | GATEASSSSAAVARR |
| **29** | ASSSSAAVARRNERE |
| **30** | SAAVARRNERERNRV |
| **31** | ARRNERERNRVKLVN |
| **32** | ERERNRVKLVNLGFQ |
| **33** | NRVKLVNLGFQALRQ |
| **34** | LVNLGFQALRQHVPH |
| **35** | GFQALRQHVPHGGAN |
| **36** | LRQHVPHGGANKKLS |
| **37** | VPHGGANKKLSKVET |
| **38** | GANKKLSKVETLRSA |
| **39** | KLSKVETLRSAVEYI |
| **40** | VETLRSAVEYIRALQ |
| **41** | RSAVEYIRALQRLLA |
| **42** | EYIRALQRLLAEHDA |
| **43** | ALQRLLAEHDAVRAA |
| **44** | LLAEHDAVRAALAGG |
| **45** | HDAVRAALAGGLLTPA |
| **46** | RAALAGGLLTPATPPS |
| **47** | AGGLLTPATPPSDEC |
| **48** | LTPATPPSDECAQPS |
| **49** | TPPSDECAQPSASPA |
| **50** | DECAQPSASPASASL |
| **51** | QPSASPASASLSCAS |
| **52** | SPASASLSCASTSPS |
| **53** | ASLSCASTSPSPDRL |
| **54** | CASTSPSPDRLGCSE |
| **55** | SPSPDRLGCSEPTSPR |
| **56** | DRLGCSEPTSPRSAY |
| **57** | CSEPTSPRSAYSSEE |
| **58** | TSPRSAYSSEESSCE |
| **59** | SAYSSEESSCEGELS |
| **60** | SEESSCEGELSPMEQ |
| **61** | SCEGELSPMEQELLD |
| **62** | ELSPMEQELLDFSSW |
| **63** | MEQELLDFSSWLGGY |

**Table B.** MASH2 peptide matrix used for T-cell immunogenicity assessment

|  | **Pool 9** | **Pool 10** | **Pool 11** | **Pool 12** | **Pool 13** | **Pool 14** | **Pool 15** | **Pool 16** |
| --- | --- | --- | --- | --- | --- | --- | --- | --- |
| **Pool 1** | 1 | 2 | 3 | 4 | 5 | 6 | 7 | 9 |
| **Pool 2** | 9 | 10 | 11 | 12 | 13 | 14 | 15 | 16 |
| **Pool 3** | 17 | 18 | 19 | 20 | 21 | 22 | 23 | 24 |
| **Pool 4** | 25 | 26 | 27 | 28 | 29 | 30 | 31 | 32 |
| **Pool 5** | 33 | 34 | 35 | 36 | 37 | 38 | 39 | 40 |
| **Pool 6** | 41 | 42 | 43 | 44 | 45 | 46 | 47 | 48 |
| **Pool 7** | 49 | 50 | 51 | 52 | 53 | 54 | 55 | 56 |
| **Pool 8** | 57 | 58 | 59 | 60 | 61 | 62 | 63 | - |

**S1 Fig.** Percentage of CD4+ T-cells producing cytokines (IFN-γ and/or TNF-α) in CB6F1 mice after stimulation with a matrix of 16 peptide pools covering the whole MASH2 sequence or with an irrelevant stimulation (Roswell Park Memorial Institute medium).

SEM, standard error of the mean.

**S2 Fig.** Multiplicity of small intestinal adenomas post-treatment. Values represent histologically confirmed adenomas in the small intestine of mice in a) Prophylactic Study 1 and b) Prophylactic Study 2 at necropsy and processed as jelly rolls.

PBS, phosphate buffer saline; recMASH2+AS15, recombinant mouse achaete scute homolog 2 protein combined with AS15 immunostimulant.

The error bars represent 95% confidence intervals.

**S3 Fig.** Multiplicity of colon adenomas post-treatment by morphological subtype. Values represent histopathologically confirmed colon a) adenomas and b) microadenomas at necropsy in mice bearing colon tumors at the time of treatment initiation (Immunotherapy Study).

PBS, phosphate buffer saline; recMASH2+AS15, recombinant mouse achaete scute homolog 2 protein combined with AS15 immunostimulant.

The error bars represent 95% confidence intervals.
